# Supplementary material for: Aberration Theory of a Flat, Aplanatic Metalens Doublet and the Design of a Meta-Microscope Objective Lens
Source: Sensors (Basel). 2023 Nov 19;23(22):9273. doi: 10.3390/s23229273 (PMC10674246; doi:10.3390/s23229273)
Supplement: Supplementary file 1 [file sensors-23-09273-s001.zip › sensors-2694408-supplementary.pdf]

ABERRATION THEORY OF FLAT APLANATIC METALENS DOUBLET AND DESIGN OF A META-MICROSCOPE OBJECTIVE LENS

S1. Derivation of the phase gradient profiles of aplanatic doublet metalens

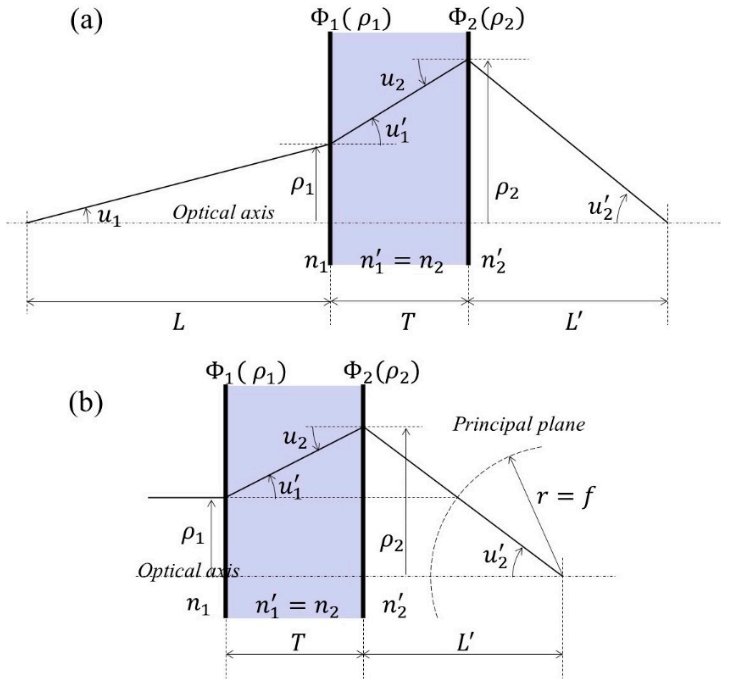

Figure S1. The Abbe aplanatic condition of a metalens doublet. Schematic diagrams of (a) a finite object and (b) an infinite object, respectively.

If $\sin u_1 / \sin u_2' = M$ or $\rho_1 / \sin u_2' = -f$ when very far distance object, for all ρ_1 is satisfied by Abbe sine condition, it becomes aplanatic system [1]. M and f are magnification and focal length of system, respectively. Therefore, when M or f is determined, the ray passing through the system can be traced according to the aplanatic condition. To achieve the Abbe sine condition, the rays from the point object on the axis located at the distance L should be focused without any aberration in the back focal length L'. The ray tracing notation and sine convention are adopted from the reference textbook by R. Kingslake [1]. The origin of coordinates is placed at the center of refracting surface. Distance along the optical axis is positive when it is measured to the right from the origin. The angles are consistent with right-handed Cartesian coordinate system; that is, a ray having a positive slope angle is considered positive [1].

Thus, the condition $n_1 \sin u_1 = M n_2' \sin u_2'$ should be satisfied for all rays by the metasurface phase gradient profiles of both sides of the substrate.

Based on the sine condition, in case of finite-finite imaging (Fig. S1(a)), the ray height positions reaching each surface are derived as $\rho_1 = -L \tan u_1$ and $\rho_2 = -L' \tan u_2' = -L' \tan\{\sin^{-1}(n_1 \sin u_1 / n_2' M)\}$. Here, n_1 and n_2' are refractive index before 1st

metasurface and after 2nd metesurface, respectively. And the difference between them is $\rho_2 - \rho_1 = T\tan u_1'$ where T is the substrate thickness (Fig. S1).

Combination of the above equations can be arranged as a relation between ρ_2 and ρ_1 as following Eq. (S1) and (S2).

For a finite distance object case,

$$\rho_2(\rho_1) = -L'\tan\{\sin^{-1}(n_1\sin u_1 / n_2'M)\} = L'\tan\left[\sin^{-1}\{n_1\sin(\tan^{-1}\{\rho_1 / L\}) / n_2'M\}\right]. \tag{S1}$$

For a very far distance,

$$\rho_2(\rho_1) = -L'\tan u_2' = L'\tan\{\sin^{-1}(n_1\rho_1 / n_2'f)\}. \tag{S2}$$

And the relation between the refractive angle u_1' after passing through the 1st surface and ρ_1 is derived as Eq. (S3) and (S4).

For a finite distance object case,

$$u_1'(\rho_1) = \tan^{-1}\left(\frac{\rho_2-\rho_1}{T}\right) = \tan^{-1}\left[\frac{L'\tan\{\sin^{-1}(n_1\sin\{\tan^{-1}(\rho_1 / L)\} / n_2'M)\} - \rho_1}{T}\right]. \tag{S3}$$

For a very far distance,

$$u_1'(\rho_1) = \tan^{-1}\left(\frac{\rho_2-\rho_1}{T}\right) = \tan^{-1}\left[\frac{L'\tan\{\sin^{-1}(n_1\rho_1 / n_2'f)\} - \rho_1}{T}\right]. \tag{S4}$$

The derived results in Eq. (S1)-(S4) are only the results obtained from the Abbe sine condition. Now we apply the generalized Snell's law to derive the required phase gradient profiles at the two surfaces. When the phase maps on the first and second metasurfaces are expressed as $\Phi_2(\rho_1)$ and $\Phi_2(\rho_2)$, respectively, and the Generalized Snell's law is applied to the surfaces, the phase gradients of the 1st and 2nd metasurfaces can be expressed as $\partial\Phi_1(\rho_1)/\partial\rho_1 = k(n_1'\sin u_1' - n_1\sin u_1)$ and $\partial\Phi_2(\rho_2)/\partial\rho_2 = k(n_2'\sin u_2' - n_2\sin u_2)$, respectively [2].

Thus, by adopting the results of Eq. (S3) and (S4), the phase gradient on each surface can be expressed as a function of ρ_1 as shown in Eq. (S5)-(S8).

For a finite distance object case,

$$\begin{aligned}\frac{\partial\Phi_1(\rho_1)}{\partial\rho_1} &= k(n_1'\sin u_1' - n_1\sin u_1)\\ &= -k\left[n_1'\sin\left\{\tan^{-1}\left[\frac{\rho_1 - L'\tan\{\sin^{-1}(n_1\sin\{\tan^{-1}(\rho_1 / L)\} / n_2'M)\}}{T}\right]\right\} - n_1\sin\{\tan^{-1}(\rho_1 / L)\}\right],\end{aligned} \tag{S5}$$

$$\begin{aligned}\frac{\partial\Phi_2\{\rho_2(\rho_1)\}}{\partial\rho_1} &= k(n_2'\sin u_2' - n_2\sin u_2)\\ &= -k\left[\frac{n_1\sin\{\tan^{-1}(\rho_1 / L)\}}{M} - n_1'\sin\left\{\tan^{-1}\left[\frac{\rho_1 - L'\tan\{\sin^{-1}(n_1\sin\{\tan^{-1}(\rho_1 / L)\} / n_2'M)\}}{T}\right]\right\}\right].\end{aligned} \tag{S6}$$

For a very far distance,

$$\begin{aligned}\frac{\partial\Phi_1(\rho_1)}{\partial\rho_1} &= k(n_1'\sin u_1' - n_1\sin u_1)\\ &= -kn_1'\sin\left\{\tan^{-1}\left[\frac{\rho_1 - L'\tan\{\sin^{-1}(n_1\rho_1 / n_2'f)\}}{T}\right]\right\},\end{aligned} \tag{S7}$$

$$\frac{\partial\Phi_2\{\rho_2(\rho_1)\}}{\partial\rho_1} = k(n_2'\sin u_2' - n_2\sin u_2)$$
$$= -k\left[\frac{n_1\rho_1}{f} - n_1'\sin\left\{\tan^{-1}\left[\frac{\rho_1 - L'\tan\{\sin^{-1}(n_1\rho_1/n_2'f)\}}{T}\right]\right\}\right] . \qquad \text{(S8)}$$

S2. Minimize tangential astigmatic blur and lateral achromatic

S2.1 Derivation of tangential astigmatic blur

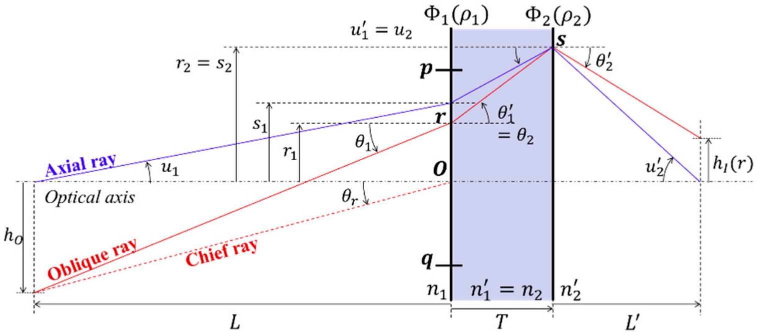

Figure S2. Schematic of oblique ray tracing to minimize tangential image blur.

Oblique and axial rays are traced to estimate and minimize tangential astigmatic blur. Fig. S2 describes the trajectories of the two rays and the chief ray passing through the center of the first metasurface, ***o***. When the system satisfies the aplanatic condition for arbitrary object distance (L), thickness of substrate (T), and back focal length (L') at magnification (M), the phase gradient of each plane can be obtained as follows.

By the Eq. (S5), the 1st phase gradient at the ***r*** position (marked in Fig. S2) through which the oblique ray passes is,

$$\Phi_1'(r_1) = -k\left[n_1'\sin\left\{\tan^{-1}\left[\frac{r_1 - L'\tan\left\{\sin^{-1}\left(n_1\sin\{\tan^{-1}(r_1/L)\}/n_2'M\right)\right\}}{T}\right]\right\} - n_1\sin\{\tan^{-1}(r_1/L)\}\right] . \qquad \text{(S9)}$$

For a very far distance, by the Eq. (S7),

$$\Phi_1'(r_1) = -kn_1'\sin\left\{\tan^{-1}\left[\frac{r_1 - L'\tan\{\sin^{-1}(n_1r_1/n_2'f)\}}{T}\right]\right\} . \qquad \text{(S10)}$$

The incident angle of oblique ray at the ***r*** position on 1st surface is,

$$\theta_1 = \tan^{-1}\{(h_O - r_1)/L\} . \qquad \text{(S11)}$$

Where h_O is height of object. For a very far distance,

$$\theta_1 = \theta_r . \qquad \text{(S12)}$$

Here, θ_r is the slope angle of the chief ray.

By the generalized Snell's law, the refracted angle after passing the 1st surface is

$$\theta_1'(r_1) = \sin^{-1}\left[\frac{1}{n_1'}\left\{\frac{\Phi_1'(r_1)}{k} + n_1\sin\theta_1\right\}\right] . \qquad \text{(S13)}$$

The height of s position at 2nd surface is expressed as a function of r_1 in Eq. (S14).

$$s_2(r_1) = r_1 + T\tan\theta_1'(r_1) . \qquad \text{(S14)}$$

By using Eq. (S1), the height on the 1st surface of the axial ray passing through the ***s*** position is derived as

$$s_1(r_1) = L\tan\left[\sin^{-1}\left\{M\frac{n_2'}{n_1}\sin\left(\tan^{-1}\frac{s_2(r_1)}{L'}\right)\right\}\right]. \qquad \text{(S15)}$$

For a very far distance, based on Eq. (S2),

$$s_1(r_1) = f\frac{n_2'}{n_1}\sin\left\{\tan^{-1}\left(\frac{s_2(r_1)}{L'}\right)\right\}. \qquad \text{(S16)}$$

From the Eq. (S6), the 2nd phase gradient at the $\boldsymbol{s}$ position through which the oblique ray passes is,

$$\Phi_2'\left[s_2\{s_1(r_1)\}\right] = -k\left[\frac{n_1\sin\{\tan^{-1}(s_1(r_1)/L)\}}{M} - n_1'\sin\left\{\tan^{-1}\left[\frac{s_1(r_1)-L'\tan\{\sin^{-1}(n_1\sin\{\tan^{-1}(s_1(r_1)/L)\}/n_2'M)\}}{T}\right]\right\}\right], \qquad \text{(S17)}$$

For a very far distance, from the Eq. (S8),

$$\Phi_2'\left[s_2\{s_1(r_1)\}\right] = -k\left[\frac{n_1 s_1(r_1)}{f} - n_1'\sin\left\{\tan^{-1}\left[\frac{s_1(r_1)-L'\tan\{\sin^{-1}(n_1 s_1(r_1)/n_2' f)\}}{T}\right]\right\}\right]. \qquad \text{(S18)}$$

From the generalized Snell's law, the refracted angle after 2nd surface is derived and organized as Eq. (S19).

$$\begin{aligned}\theta_2'(r_1) &= \sin^{-1}\left[\frac{1}{n_2'}\left\{\frac{\Phi_2'\left[s_2\{s_1(r_1)\}\right]}{k} + n_2\sin\theta_2\right\}\right]\\ &= \sin^{-1}\left[\frac{1}{n_2'}\left\{\frac{\Phi_2'\left[s_2\{s_1(r_1)\}\right]}{k} + n_1'\sin\theta_1'(r_1)\right\}\right]\\ &= \sin^{-1}\left[\frac{1}{n_2'}\left\{\frac{1}{k}\left(\Phi_1'(r_1) + \Phi_2'\left[s_2\{s_1(r_1)\}\right]\right) + n_1\sin\theta\right\}\right]\end{aligned}. \qquad \text{(S19)}$$

The oblique ray height at the back focal plane is

$$\begin{aligned}h_l(r_1) &= r_1 + T\tan\theta_1'(r_1) + L'\tan\theta_2'(r_1)\\ &= r_1 + T\tan\left[\sin^{-1}\left\{\frac{1}{n_1'}\left[\frac{1}{k}\Phi_1'(r_1) + n_1\sin\{\theta_1(r_1)\}\right]\right\}\right]\\ &\quad + L'\tan\left[\sin^{-1}\left\{\frac{1}{n_2'}\left[\left\{\frac{1}{k}\left(\Phi_1'(r_1) + \Phi_2'\left[s_2\{s_1(r_1)\}\right]\right) + n_1\sin\theta_1(r_1)\right\}\right]\right\}\right]\end{aligned}. \qquad \text{(S20)}$$

$h_l(r_l)$ in Eq. (S20) implies the magnitude of the amount of the tangential astigmatic blur of the system when the point $\boldsymbol{r}$ is moved to the margin of the 1st surface so that r_l becomes the semi-diameter of the 1st surface.

S2.2 Finite-to-finite design of a meta-microscope objective lens

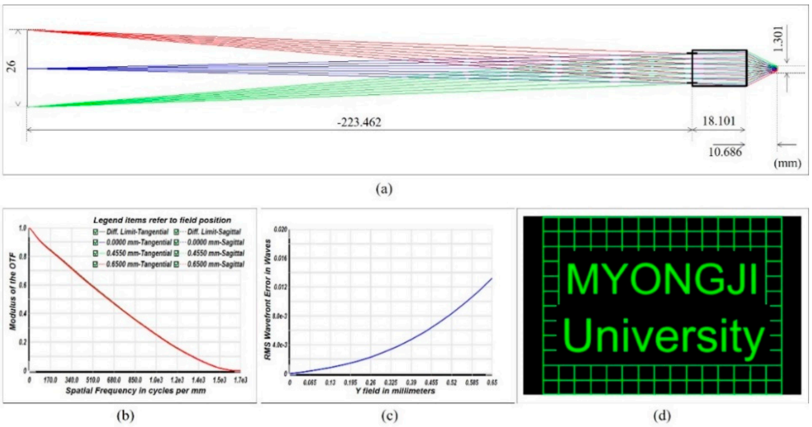

Figure S3. Finite-to-finite design results. (a) Layout of optimized aplanatic meta-microscope objective lens. (b) MTF plot. (c) RMS wavefront error plot. (d) Image simulation result.

Using the proposed semi-analytic optimization solution, the authors also design a meta-microscope objective lens that satisfies NA 0.5, magnification 20X, and field of view ±0.65 mm at 0.532 um wavelength (Fig. S3(a)). The system is designed as an finite to finite conjugate, and the combination of phase gradient that can minimize image blur was numerically found with object distance (L), back focal length (L') and substrate thickness (T) as variables. The optimized phase gradients are fitted as less than than MSE (Mean Squared Error) 1.9 E-11 in function $\Phi'(\rho)=\sum_{n=1}^{6} A_{2n-1}\rho^{2n-1}$ when the object distance, thickness of substrate and back focal length are -223.462, 15.470 and 8.681 mm, respectively. And the performance is verified with the Binary 2 surface which adds phase to the ray according to the following polynomial expansion: $\Phi(\rho)=\sum_{n=1}^{m} A_{2n}\rho^{2n}$, provided by Zemax Opticstudio.

With a paraxial magnification of -0.05X, the system has diffraction limited performance and RMS wavefront error of less than λ/30 for entire fields (Fig. S3(b) and S3(c)). Image simulation result in Fig. S3(d) also verifies its excellent near-diffraction limited imaging performance at the design wavelength (0.532 um).

S2.3 Derivation of compensation of lateral chromatic aberration

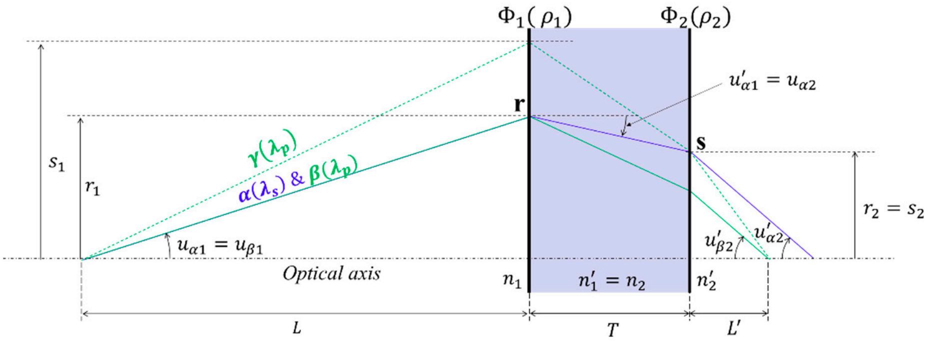

Figure S4. Scheme of the ray tracing for the lateral achromatic system at two different wavelengths. The figure describes that the back focal point is separated according to the wavelength since the lens is set to be laterally achromatic rather than longitudinally achromatic. The three different axial ray trajectories (marked as α, β, and γ) are depicted in the figure. α is the axial ray with the secondary blue wavelength (λ_s), passing through the point r and s at the first and second metasurfaces and reach the back focal point. On the other hand, β is the axial

ray passing through the r point and goes to the different back focal point due to the difference of wavelength (wavelength of λ_p). γ is the axial ray of the primary wavelength (λ_p) passing the s point at the second metasurface and focused at the same focal point with the ray β.

If the effective focal lengths and magnifications at the two wavelengths (the primary and secondary wavelengths) are equal, the lens is called 'laterally achromatic'. Effective focal lengths are calculated based on paraxial approximation. Therefore, using the paraxial approximation, the phase profile satisfying the aplanatic condition for the primary wavelength (λ_p) and the lateral achromatic condition for the secondary wavelength (λ_s) can be derived.

For a finite distant object case with a fixed magnification (M), the the aplanatic phase gradient conditions of the two surfaces proposed in section S1 can be expressed as Eq. (S21) - (S31) by the first order approximation.

$$\frac{\partial\phi_1(\rho_1)}{\partial\rho_1}=-\frac{2\pi\rho_1}{\lambda_p L}\left\{\frac{n_1'}{T}\left(L-\frac{n_1 L'}{n_2' M}\right)-n_1\right\}, \quad \text{(S21)}$$

$$\frac{\partial\phi_2\{\rho_2(\rho_1)\}}{\partial\rho_1}=-\frac{2\pi\rho_1}{\lambda_p L}\left\{\frac{n_1}{M}-\frac{n_1'}{T}\left(L-\frac{n_1 L'}{n_2' M}\right)\right\}, \quad \text{(S22)}$$

$$\rho_2(\rho_1)=\frac{L' n_1 \rho_1}{L n_2' M}. \quad \text{(S23)}$$

For a very far object,

$$\frac{\partial\phi_1(\rho_1)}{\partial\rho_1}=-\frac{2\pi\rho_1}{\lambda_p}\left\{\frac{n_1'}{T}\left(1-\frac{n_1 L'}{n_2' f}\right)\right\}, \quad \text{(S24)}$$

$$\frac{\partial\phi_2\{\rho_2(\rho_1)\}}{\partial\rho_1}=-\frac{2\pi\rho_1}{\lambda_p}\left\{\frac{n_1}{f}-\frac{n_1'}{T}\left(1-\frac{n_1 L'}{n_2' f}\right)\right\}, \quad \text{(S25)}$$

$$\rho_2(\rho_1)=\frac{L' n_1 \rho_1}{n_2' f}. \quad \text{(S26)}$$

As described in Fig. S4, when a paraxial ray α with a wavelength λ_s, the angle of refraction passing through the point **r** of the 1st metasurface, $u_{\alpha1}'$, is determined according to Eq. (S3) and (S4) and generalized Snell's law. That is,

$$u_{\alpha1}'=-r_1\left[\lambda_s/\lambda_p\left\{n_p(L-L'/M)/T-1\right\}+1\right]/(Ln_s). \quad \text{(S27)}$$

Where r_1 is height of ***r*** position. The refractive indices (marked as n_2 in Fig. S5) of the substrate are n_p and n_s at the two wavelengths (λ_p and λ_s), respectively. Therefore, the ray height passing through **s** position is,

$$r_2(r_1)=s_2(r_1)=r_1+Tu_{\alpha1}'=r_1-T\frac{r_1}{Ln_s}\left[\frac{\lambda_s}{\lambda_p}\left\{\frac{n_p}{T}\left(L-\frac{L'}{M}\right)-1\right\}+1\right]. \quad \text{(S28)}$$

Applying the generalized Snell's law with wavelength at the each side of the surface, the relation between the two phase gradient maps are derived as follows:

$$(\lambda_s/2\pi)\left\{\frac{\partial\phi_1}{\partial\rho_1}(r_1)+\frac{\partial\phi_2}{\partial\rho_1}\left[s_2\{s_1(r_1)\}\right]\right\}=u_{\alpha2}'-u_{\alpha1}. \quad \text{(S29)}$$

In addition to the setting of $u_{\alpha1}=u_{\beta1}$, as the system has the same magnification ($u_{\alpha2}'=u_{\beta2}'$) for the wavelengths λ_s and λ_p, the lateral achromatic design condition without sacrificing the aplanatic condition at the λ_p is derived as a relation between T, M, L, and L' for a finite-finite case (Eq. (S30).

$$\frac{\lambda_s}{\lambda_p}\left[\left\{\frac{n_p}{T}\left(L-\frac{L'}{M}\right)-1\right\}+\frac{M}{L'}\left\{L-\frac{T}{n_s}\left[\frac{\lambda_s}{\lambda_p}\left\{\frac{n_p}{T}\left(L-\frac{L'}{M}\right)-1\right\}+1\right]\right\}\left\{\frac{1}{M}-\frac{n_p}{T}\left(L-\frac{L'}{M}\right)\right\}\right]-\left(\frac{1-M}{M}\right)=0. \quad \text{(S30)}$$

For a very far distance object, the condition for lateral achromatic and aplanatic at the wavelength of λ_p is changed as Eq. (S31).

$$\frac{\lambda_s}{\lambda_p}\left[\left\{\frac{n_p}{T}\left(1-\frac{L'}{f}\right)\right\}+\frac{f}{L'}\left\{1-\frac{\lambda_s n_p}{\lambda_p n_s}\left(1-\frac{L'}{f}\right)\right\}\left\{\frac{1}{f}-\frac{n_p}{T}\left(1-\frac{L'}{f}\right)\right\}\right]-\frac{1}{f}=0\,. \tag{S31}$$

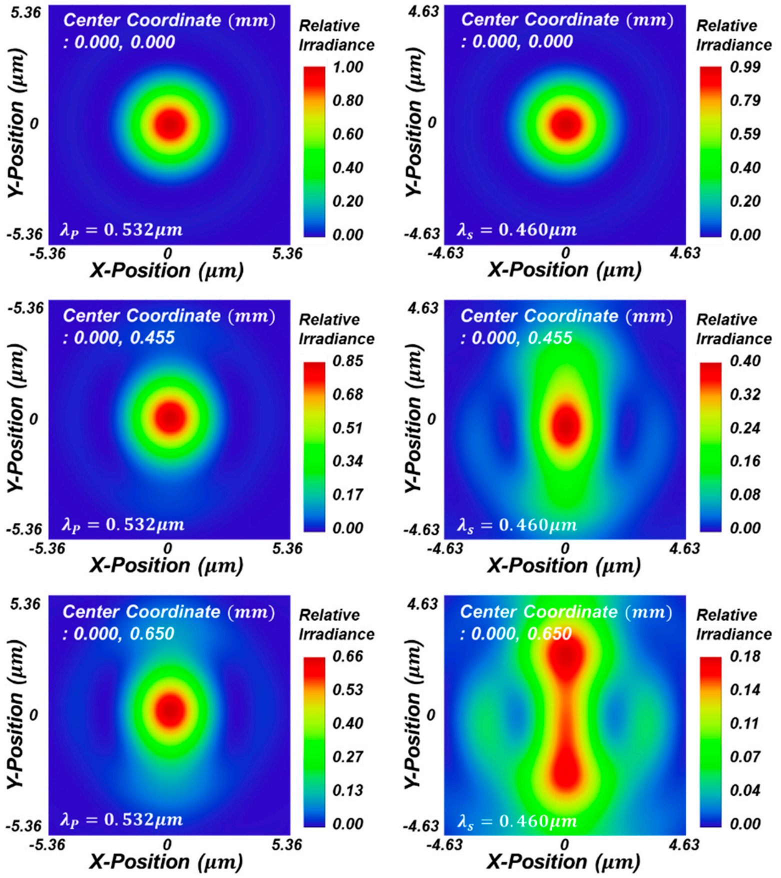

Figure S5. Simulated point spread function (PSF) intensity profiles of lateral achromatic doublet in XY plane at each focal plane respectively.

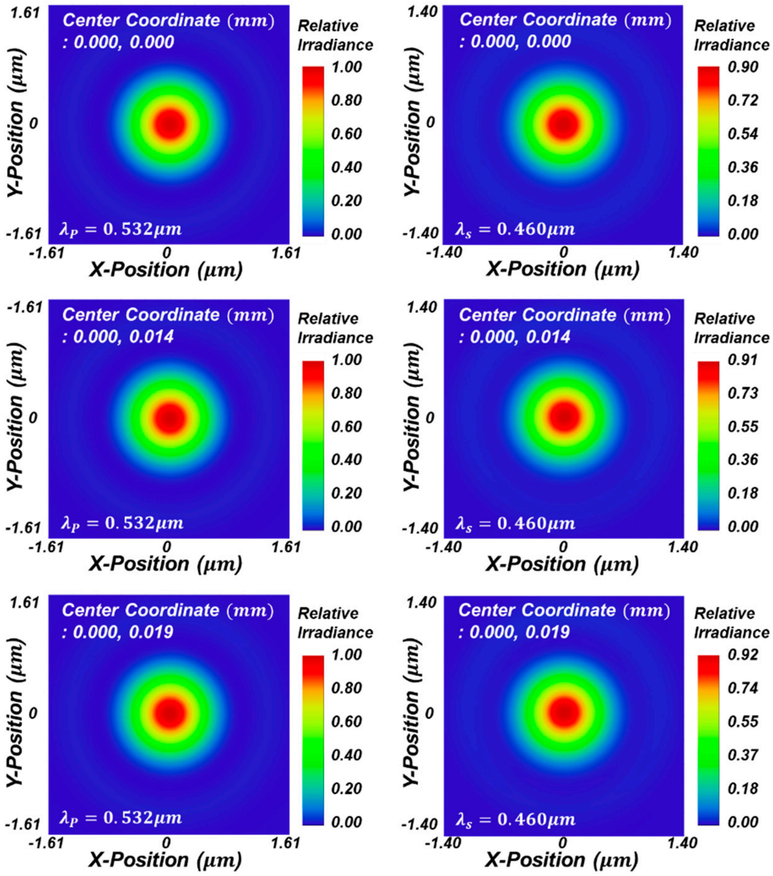

Figure S6. Simulated point spread function (PSF) intensity profiles of lateral achromatic doublet with small size in XY plane at each focal plane respectively.

S3. MATLAB codes for semi-analytical design of the proposed doublet phase profiles

S3.1 Condition for monochromatic aplanatic doublet

```
clear; clc; close all;

%%%%Input Values%%%%
%%%%%%%%%%%%%%%%%%%%
f=10; % effective focal length (mm)
L=8.681; % back focal length (mm)
Lam=0.000532; % wavelength (mm)
T=15.47; %substrate thickness (mm)
n=1.5468694858; %quartz's refractive index at 532nm wavelenght
D=10; % aperture Diameter (mm)
S=0.01; % Sampling pitch of aperture (mm)
%%%%%%%%%%%%%%%%%%%%%
%%%%%%%%%%%%%%%%%%%%%

syms x
k=2*pi/Lam; %wavenumber
u2(x)=asin(-x/f); %incident angle at focal plane
u1(x)=atan(-(tan(u2)*L+x)/T); % refractive angle after 1st surface
dy1(x) = k*n*sin(u1); %phase gradient of 1st surface
x2(x)=x+T*tan(asin(dy1/(n*k)));%radius of 2nd surface.
dy2(x)=k*(-x/f-n*sin(u1));%phase gradient of 2nd surface
x=-D/2:S:D/2; %radius range of 1st surface.
ans1=double(dy1(x)); %ploted phase gradient of 1st surface
ansx2=double(x2(x));%plotted radius of 2nd surface
ans2=double(dy2(x));%plotted phase gradient of 2nd surface

% First surface's gradient
figure
plot(x, ans1, 'b')
xlabel('r1')
ylabel('Phase Gradient')
title('1st surface')
% Second surface's gradient
figure
plot(ansx2, ans2, 'r')
xlabel('r2(r1)')
ylabel('Phase Gradient')
title('2nd surface')
```

S3.2 Minimization of tangential astigmatic blur

```
clear; clc; close all;

%%%%Input Values%%%%
%%%%%%%%%%%%%%%%%%%%
f=10; %effective focal lenghth (mm)
fov=1.3; %full FoV in (mm)
ENPD=5; %Entrance pupil diameter
Lam=0.000532; % wavelength (mm)
n=1.5468694858; %quartz's refractive index at 550nm wavelenght
T1=10;%minimum Thickness (mm)
T2=20;%maximum Thickness (mm)
L1=5;%minimum Back focal lenght (mm)
L2=15;%maximum Back focal lenght (mm)
S=0.1;%sampling pitch of Thickness & Backfocal lenght (mm)
```

```
%%%%%%%%%%%%%%%%%%%%%
%%%%%%%%%%%%%%%%%%%%%

k=2*pi/Lam; %wavenumber
v=atan((fov/2)/f); % half FoV in (rad)
field=rad2deg(v); % half FoV in (degree)
P=ENPD/2; %maximum radius of upper aperture stop
Q=-ENPD/2; %maximum radius of lower aperture stop
[T,L]=meshgrid(T1:S:T2,L1:S:L2); %sampling of Thickness & Backfocal
length

P1=-k*n*sin(atan((P-L.*tan(asin(P/f)))./T)); %1st phase gradient at P
Pvv=asin((P1/k+sin(v))/n); %diffracted marginal angle at P after 1st
surf.
PP=P+T.*tan(Pvv); %The position on the second surface of the marginal
field passing through point P.
RP=f*sin(atan(PP./L)); %The position of the first surface to
calculate the gradient at the position of PP.
P2=-k*(RP/f-n*sin(atan((RP-L.*tan(asin(RP/f)))./T)));%2nd phase
gradient at PP
Pvvv=asin(P2/k+n*sin(Pvv)); %The refractive angle after the second
surface of the marginal field passing through point P.
PPP=PP+L.*tan(Pvvv); %The height at the focal plane of the marginal
field passing through point P.

Q1=-k*n*sin(atan((Q-L.*tan(asin(Q/f)))./T)); %1st phase gradient at Q
Qvv=asin((Q1/k+sin(v))/n); %diffracted marginal angle at Q after 1st
surf.
QQ=Q+T.*tan(Qvv); % The position on the second surface of the
marginal field passing through point Q.
RQ=f*sin(atan(QQ./L)); %The position of the first surface to
calculate the gradient at the position of QQ.
Q2=-k*(RQ/f-n*sin(atan((RQ-L.*tan(asin(RQ/f)))./T)));%2nd phase
gradient at PP
Qvvv=asin(Q2/k+n*sin(Qvv)); %The refractive angle after the second
surface of the marginal field passing through point Q.
QQQ=QQ+L.*tan(Qvvv); %The height at the focal plane of the marginal
field passing through point Q.

%Plot of Blur of Marginal Field.
B=abs(QQQ-PPP);
figure, contourf(T,L,B,100, 'linestyle','none'),colorbar;
```

S3.3 Extension to lateral achromatic design

```
clear; clc; close all;

%%%%Input Values%%%%
%%%%%%%%%%%%%%%%%%%%%
f = 10; % effective focal length (mm)
wP = 0.000532; % primary wavelength (mm)
wS = 0.000460; % secondary wavelength (mm)
nP = 1.5468694858; % primary wavelength's refractive index of quartz
nS = 1.5516044205; % secondary wavelength's refractive index of
quartz
T1=15.5;%minimum Thickness (mm)
T2=20;%maximum Thickness (mm)
S=0.01;%sampling pitch of Thickness(mm)
%%%%%%%%%%%%%%%%%%%%%
```

```
%%%%%%%%%%%%%%%%%%%%%

T_values = T1:S:T2; % T values to iterate over
L_values = zeros(size(T_values)); % initialize L_values array
syms L;
for i = 1:length(T_values)
    T = T_values(i);

    A = 0;
    A = wS/wP.*(nP./T.*(L./f-1)-f./L.*(1+nP./nS*wS/wP.*(L./f-
1)).*(1./f+nP./T.*(L./f-1)))+1/f; %Equation (21)

    L_solve = solve(A==0, L, 'Real', true); % solve for L values
    L_values(i) = min(double(L_solve(L_solve > 0))); % save the first
positive L value for current T
end

%Plot of Backfocal lenght value according to Thickness
plot(T_values, L_values,'b'); % plot T vs L
xlabel('T');
ylabel('L');
title('Combination of T&L');
```

S4. Meta-atom simulations

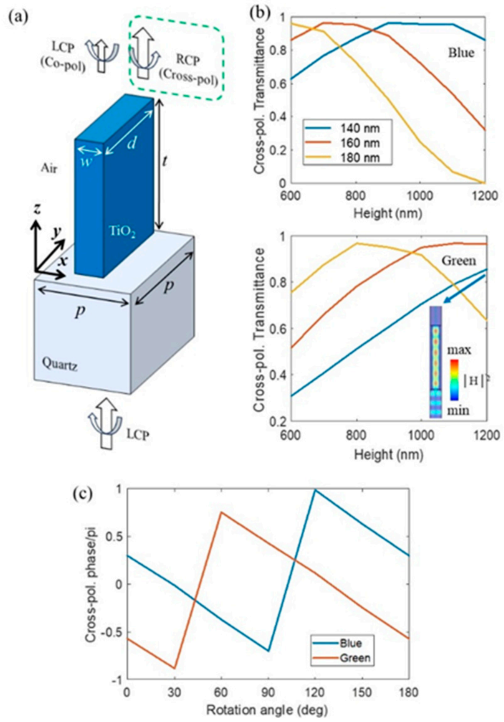

Figure S7. Electromagnetic simulation results for a design example of a TiO_2 nanofin meta-atom. (a) Schematic diagram of a TiO_2 nanofin unitcell for geometric phase modulation located on Quartz substrate. p and w are fixed to be 210 and 60 nm, respectively. Cross-polarized transmittance for the (b) blue (0. 460 μm, upper figure) and (c) green (0.532 μm, lower figure) according to the change of t (height). The legends in (b) denote the values of d and the inset

figure in the lower figure in (b) describe the magnetic field intensity profile in the *xz* plane containing the center of the nanofin. (c) Numerical verification of geometric phase modulation according to the nanofin rotation angle in the *xy* plane.

Electromagnetic simulation results of high-efficiency nanofin unitcell for geometric phase modulation is provided as seen in Fig. S7(a). For the simulations, COMSOL Multiphysics 5.6 RF solver is used and the optical property of TiO_2 is quoted from the literature [3]. Local periodic approximation, the basic and conventional method to design nanostructures for phase-gradient metasurface, is assumed by neglecting near-field coupling between adjacent nanofins. The optimal conditions are searched through some parameter sweeps when the fast axis nanofin length (w) and unitcell period (p) are fixed as the constant values. Fig. S7(b) shows that the height (t) of 1200 nm and the slow axis length (d) of 140 nm can be the optimal choice for the most efficient nanofin design in the suggested simulation results. For the chosen values of p (210 nm), d (140 nm), w (60 nm), and t (1200 nm), it is found that the theory of geometric phase for full phase modulation by 180 deg rotation shows good agreement with numerical results at the both wavelengths suggested in Fig. S7 (c). And there is enough room for improving cross-polarization transmittance for the two design wavelengths, if advanced numerical optimization based on numerous repetitive simulations is adopted.

References

1. Kingslake, R.; Johnson, B. Lens Design fundamentals, 2nd ed.; Academic Press: Cambridge, MA, USA, 2009.
2. Yu, N.; Genevet, P.; Kats, M. A.; Aieta, F.; Tetienne, J. P.; Capasso, F.; Gaburro, Z. Light propagation with phase discontinuities: generalized laws of reflection and refraction. Science 2011, 334, 333-337.
3. Khorasaninejad, M.; Chen, W.T.; Devlin, R.C.; Oh, J.; Zhu, A.Y.; Capasso, F. Metalenses at visible wavelengths: Diffraction-limited focusing and subwavelength resolution imaging. Science 2016, 352, 1190–1194.
